# Development of Combination HIV Prevention Programs for People Who Inject Drugs through Government and Civil Society Collaboration in the Russian Federation

**DOI:** 10.1155/2012/874615

**Published:** 2012-11-29

**Authors:** M. V. Volik, G. A. Karmanova, E. B. Berezina, T. F. Kresina, R. G. Sadykova, L. N. Khalabuda, F. Z. Fattakhov

**Affiliations:** ^1^Center for Social Development and Information (Population Services International/Russia), 125315 Moscow, Russia; ^2^Institute for Public Health and Health Management, First Moscow State Medical University, 119021 Moscow, Russia; ^3^Division of Pharmacologic Therapies, Center for Substance Abuse Treatment, Substance Abuse and Mental Health Services Administration, Rockville, MD 20857, USA; ^4^Division for Anti-Drug Work Coordination, Cabinet of Ministers of the Republic of Tatarstan, Kazan, 420060 Republic of Tatarstan, Russia; ^5^Public Organization “Prevention and Initiative”, Kazan, 420044 Republic of Tatarstan, Russia; ^6^Republican Drug Treatment Hospital, Kazan, 420082 Republic of Tatarstan, Russia

## Abstract

Population Services International (PSI) has worked collaboratively with several government institutions of the Russian Federation to develop and implement a model program to access health services for individuals who are opioid dependent, including those with HIV infection. Through the development of partnership agreements between government organizations (GOs) and non-government organizations (NGOs), a model of the continuum of care has been developed that identifies a Recommended Package of HIV Prevention Services for Injecting Drug Users (RPS-IDU). The implementation of the RPS-IDU in the Russian Federation offers a model for other countries with HIV epidemics associated with injection drug use. This paper will describe the model program and its implementation in one of the pilot program regions.

## 1. Introduction

Globally, the trafficking and subsequent use of addictive substances is widespread [1, 2]. Vulnerability to drug dependence, particularly heroin, can occur rapidly for injection drug users and is behaviorally complex as a function of biological, psychological, and environmental interactions and influences. In the Russian Federation heroin, trafficked from Central Asia, is readily available for use with dependence manifested as a chronic relapsing brain disease [2]. The clinical diagnosis of opioid dependence, according to the tenth revision of the International Statistical Classification of Diseases and Related Health Problems (ICD-10), is not based on the quantity of drug used, but the maladaptive patterns of drug use, as well as cognitive, behavioral, and physiological symptoms including any significant consequences related to drug use [3]. International treatment guidelines promote effective treatment programs that have multiple components offering an array of services and pharmacotherapies that address the cognitive, behavioral, physiological, and social aspects of opioid dependence [4]. Opioid treatment programs also need to address the medical comorbidities associated with injection drug use, most significantly Human Immunodeficiency Virus (HIV).

The World Health Organization (WHO) has identified a comprehensive set of interventions for HIV prevention, care, and treatment for injection drug users [5]. They include needle and syringe programs; drug dependence treatment; targeted information, education, and communication for people who use drugs; HIV testing and counseling; HIV care and treatment; safe and effective condom use; detection and management of sexually transmitted infections; prevention and treatment of viral hepatitis; tuberculosis prevention, diagnosis, and treatment. In 2010, the Joint United Nations Program on HIV/AIDS also identified a *combination prevention* approach that relies on the evidence-informed, strategic, simultaneous use of complementary behavioral, biomedical, and structural prevention strategies [6].

### 1.1. Opioid Dependence and the HIV Epidemic in the Russian Federation

Because of the large amount of opioids trafficked from Central Asia through the Russian Federation, inexpensive heroin is available and readily accessible. Data from 2009 indicate that more than 550,000 people in the Russian Federation are officially registered as drug users [7] and 567,558 people are living with HIV infection [8]. Although the estimated number of people who inject drugs (PWIDs) varies among different international and national studies, drug use and HIV infection are significantly underreported [9]. *The World Drug Report 2010* estimates that approximately 1.6 million people in the Russian Federation use opioids [2], which represents 1 percent of the total Russian population. In some regions of the Russian Federation, it is estimated that the HIV prevalence in the opioid dependent populations can reach as high as 75% [2]. Official statistics for the Russian Federation indicate that from 1987 to 2008 approximately 80% of all HIV infections were associated with injection drug use and opioid dependence [10]. Thus, the Russian Federation has a serious dual epidemic of opioid dependence and HIV infection.

The federal health care system in the Russian Federation is a post-Soviet system that is publically owned and financed with the government managing resource allocation, and health care professionals are government employees [11]. As a centrally planned and managed system, medical services are free and directed from Moscow through government decrees called “prikaz.” The health care system focuses on the treatment of prevailing acute diseases and gives priority to inpatient treatment of acute conditions rather than chronic conditions [11]. The focus on inpatient care has resulted in a vertical or “stove-piped” hospital-based medical specialty system of separate services delivered by subspecialty trained physicians [11]. Thus, patients who need narcology services (drug treatment services) are required to seek those services at a narcological hospital where the services are specifically and strictly related to addiction treatment. As a parallel system, the Russian government developed regional AIDS Centers to provide HIV-specific services for patients with HIV infection.

HIV prevention programs for PWID in the Russian Federation provide only limited services. Current HIV programs are focused mostly on treatment with limited funding for prevention. While Russian federal statistics demonstrate that HIV infection is a public health problem for PWID, government stakeholders have not recognized the importance of HIV prevention services for PWID in the context of larger efforts to limit the spread of HIV infection. These larger efforts must include an integrated program of HIV prevention that provides a continuum of care for opioid dependence to reduce HIV transmission and new incident infections.

## 2. Methods: Developing the Recommended Package of HIV Prevention Services for Injection Drug Users (RPS-IDUs)

PSI responded to the identified gap by developing a specific health care program utilizing the current federal system of care but comprising a full continuum of care to PWID. In 2010, PSI launched a new five-year program, HIV Prevention for At-Risk Populations in Russia, with funding from USAID. PSI is implementing this program in cooperation with GBCHealth, an international coalition of more than 200 member business companies and organizations. Based on epidemiological need, previous program experience and the regional political will, the cities of Saint Petersburg, Kazan (Republic of Tatarstan), Barnaul (Altay Krai), and Samara Oblast were selected as regional sites for program implementation. Primary target groups of this program are PWID and PWID subpopulations, including people who are engaged in providing sexual services, released prison inmates, men who have sex with men (MSM), and people with HIV infection and recovering PWID. In addition, the program works with sexual partners and families of PWID to provide needed services. Separate program activities (education on medical and nonmedical issues, sensitization on specific needs of key vulnerable populations) are designed for service providers who work at nongovernmental and public organizations, medical professionals and social workers, and official representatives of the healthcare system at regional and federal levels.

PSI assembled a group of government stakeholders, public health and medical experts, and NGO practitioners to develop a Recommended Package of Services for Injecting Drug Users (RPS-IDUs) as part of the USAID-funded program. The RPS-IDU is based on WHO/PEPFAR-recommended best practices, acculturated to the Russian context, and informed by the barriers faced by other programs implemented in Russia to date. The RPS-IDU is also based on research conducted in areas where dual HIV and opioid dependence epidemics coexist, which document that integrated or colocated health care services are most effective for PWID in accessing and remaining in care and treatment [12]. Accordingly, the RPS-IDU focuses on developing an integrated system of services provided by NGOs and government-funded facilities and incorporates evidence-based services that can be implemented and are acceptable to both patients and health care providers. To this end, partnerships were developed with the National Research Center on Addictions, the Federal AIDS Center, and experts from other federal and regional healthcare institutions. 

**Figure 1:**
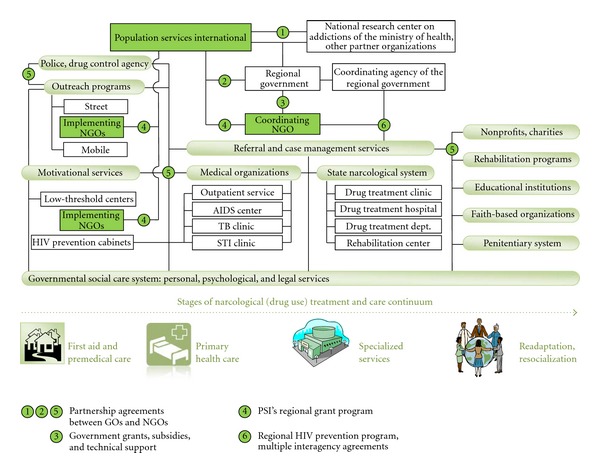
Implementation of the RPS-IDU Model at the regional level.

General principles of the RPS-IDU model development at the regional level are illustrated in Figure 1. The model reflects a 4-stage healthcare service provision system that includes premedical care, primary healthcare (outpatient services), specialized (inpatient) services, and rehabilitation programs with the critically important readaptation and resocialization services that support patients recovering from drug dependence. The RPS-IDU model is supported through close collaboration between PSI and the National Research Center on Addictions (NRCAs) of the Ministry of Health and Social Development of the Russian Federation at the federal level. Government and nongovernment organizations at the regional level are carefully coordinated by a designated agency of the regional government.

In comparison with medical facilities like clinics and hospitals, premedical care for PWID in Russia is mostly supported through projects funded by international donors. This stage includes such services as street and mobile outreach, and low-threshold (drop-in) centers that reach out to PWID and offer information, basic hygiene education, and other assistance, all designed to increase motivation for further involvement with the program. In addition, some medical facilities can open prevention cabinets, a separate unit within their infrastructure, that do not require routine registration and ID to get medical services like HIV counseling and testing or STI testing and treatment. Through a system of grants, PSI supported initial collaboration between NGOs and state medical and social institutions to offer premedical services at this stage.

The RPS-IDU model has been developed and piloted in three Russian regions, including Kazan (Republic of Tatarstan), Samara Oblast, and Barnaul (Altay Krai). St. Petersburg played a separate role of a methodological hub to demonstrate a possible prototype of the RPS-IDU model to other regions. In each region, the pilot program is supported and monitored through a team of medical experts, government representatives, and NGOs. 

## 3. Results: The Model RPS-IDU Program—A Case Study of Kazan, Republic of Tatarstan

Over the last decade, state-funded medical assistance for PWID in Kazan has undergone an extensive change from isolated individual projects to a comprehensive HIV prevention model. Under the leadership of the Antidrug Commission in the Republic of Tatarstan, a new HIV prevention program was developed through the cooperation between governmental and nongovernmental organizations. Donor-supported NGO participation was facilitated through the USAID-funded program HIV Prevention for At-Risk Populations in Russia. The program's goal is to maximize HIV prevention service coverage of PWID using the WHO/PEPFAR-Recommended Package of HIV Prevention Services acculturated to the Russian context. 

To pilot the RPS-IDU program in Kazan, a Regional Expert Working Group (REWG) comprising members of state institutions and local NGOs was formed in May 2011. The REWG with PSI support assessed the expertise and experience of civil society organizations located in Kazan in developing a collaborative model of HIV prevention service delivery with state institutions. The assessment also documented the current risky behaviors of PWID and their access to and utilization of medical services.

The REWG has created a regional HIV prevention model that defines and provides a range of services for PWID. Components of the model include the following: (1) established NGOs deliver outreach and HIV prevention services to PWID and their codependants, including referrals to state medical institutions; (2) TB, sexually transmitted infections (STIs) clinics, and AIDS center provide diagnostic and treatment services; (3) state social services, NGOs, and PLHIV activists collaboratively organize social support programs.

At the implementation level, the program works with the following partners in Kazan. 

(1) Public organization:* “Prevention and Initiative” *of the Republic of Tatarstan plays a key coordinating role in organizing outreach and case management activities for PWID in Kazan. The organization provides stationary and mobile low-threshold services to PWID and leads the network of NGO partners in the region. With support from the United Nations Office on Drugs and Crime (UNODC) and local government, the organization opened the low-threshold center “Ostrov” (“Island”) for PWID in May 2009. The center offers a range of low-threshold services, which means that clients do not need to show ID or referral slips, or abstain from drug use in order to receive services offered by the center. The center's skilled psychologists, health providers, and peer counselors provide a number of services, including substance abuse counseling, HIV counseling, referrals to medical and social services, and HIV case management. Clients of the center can also receive informational materials on HIV, STIs, rehabilitation clinics and medical services, and access to personal hygiene services. In 2011, *Prevention and Initiative* received a large government grant to support activities previously supported with donor funding, including funding from the HIV Prevention for At-Risk Populations in Russia program.

(2) Autonomous charitable nonprofit organization: *“New Century”* has been working on the prevention of HIV infection since 1998. The organization's priorities in HIV prevention include ensuring universal access to prevention, treatment, care, and support for such vulnerable populations as sex workers, men who have sex with men, PWID, and migrants. The organization is based on the Republican STI Clinic and maintains close contacts with various ministries and agencies. The organization is staffed with highly professional specialists who have academic backgrounds and affiliations, as well as a significant practical experience.

(3) Social rehabilitation center for youth: *“Wind Rose”* opened in 2002 to assist the social rehabilitation of drug users by providing 12-step Narcotics Anonymous rehabilitation. The Center was founded with support of the NGO *“Faith,”* an organization of close relatives and parents of people who use drugs. Staff of the center include volunteer exusers who have successfully completed a full course of rehabilitation. The program is funded by the city of Kazan. The center provides a social and psychological support following drug treatment at the governmental narcological and rehabilitation institutions of Kazan and the region. Services include a forum where recovering drug users receive education in a drug-free environment on coping with relapse and developing sobriety skills.* “Wind Rose”* is an essential referral point for the HIV Prevention for At-Risk Populations in Russia program clients who have received medically assisted treatment at government drug treatment centers.

(4) Nongovernmental organization: *Social Bureau “Phoenix” *was established in 2011. The NGO uses case management to help at-risk populations adapt to a normal life within society. *“Phoenix”* also engages in the prevention of infectious diseases, including tuberculosis, STIs, HIV infection, and viral hepatitis. The organization primarily targets prison inmates at the pre- and postrelease stages, many of whom have a history of injecting drug use and are living with HIV. *“Phoenix”* partners with the Center for Social Adaptation for the Homeless and Unemployed of the Republican Ministry of Labor and Social Protection, which provides social, medical, economic, and legal services to homeless people and people in difficult life situations.

By May 2012, 3057 PWID in Kazan, or approximately 46% of the total estimated number of PWID population in Kazan (6,600), had received more than one service included into the RPS-IDU approach. Noting the unmet need, the government recently allocated more funding for NGO service programs [13]. The model comprehensive HIV prevention program for PWID in Kazan provides accessible, client-friendly, quality prevention services in collaboration with state institutions. Through increasing state support, civil society organizations can consolidate, optimize, and maximize the delivery of cost-effective HIV prevention services. 

## 4. Discussion

Given the diversity of health and social problems that PWID and their sexual partners experience, combination prevention is the most effective approach for preventing HIV infection and other blood-borne and sexually transmitted infections in PWID, their sexual partners, and their communities [14]. This approach should include a variety of medical and social services that can readily adapt to changing needs and circumstances. It is also important that the services are carefully coordinated at the local level [14]. 

Program implementation to date has fostered a growing engagement of federal and regional government authorities in PWID-focused NGO activities and increased coordination between medical services and NGOs. In all regions, NGOs that implement the program in cooperation with PSI receive increased technical and financial support from local administrations through contracts and subsidies. Both NGOs and government organizations have been promoted to establish intra- and interregional partnerships that support the sharing of experiences, best practices, and information; communication within partnerships; preparation and dissemination of educational materials. Additional visits are organized for managers of outreach and case management programs to St. Petersburg where the program supports a methodological hub to provide first-hand experience through on-site training programs. The heightened participation of the federal government promotes the service program's sustainability.

Collaboration between government and civil society to develop the RPS-IDU has resulted in critical buy-in for the provision of HIV prevention services to at-risk populations and has created effective linkages across regions, sectors, and medical fields. The implementation of the RPS-IDU in Russia provides a blueprint for programs for PWID in other countries with similar HIV prevention needs.
